# Improvement of the sepsis survival rate by adenosine 2a receptor antagonists depends on immune regulatory functions of regulatory T-cells

**DOI:** 10.3389/fimmu.2022.996446

**Published:** 2022-09-06

**Authors:** Teng Zhang, Jie Zhao, Jingnan Fu, Guibing Chen, Tao Ma

**Affiliations:** ^1^ Department of General Surgery, Tianjin Medical University General Hospital, Tianjin, China; ^2^ Department of Intensive Care Unit, Tianjin Medical University General Hospital, Tianjin, China

**Keywords:** septic peritonitis, adenosine 2A receptor, regulatory T-cells, neutrophils, bacterial clearance

## Abstract

Adenosine shows a significant immunosuppressive effect in sepsis *via* binding to the adenosine 2a receptor (A2aR). Both genetic deletion and pharmacological inhibition of the A2aR may improve survival in sepsis. However, available research on this protective mechanism is quite limited. We used an A2aR antagonist (ZM241385) to treat a cecal ligation and puncture model of normal mice or regulatory T-cell (Treg)-depletion mice and found that the protective effect of ZM241385 is dependent on Tregs. Mechanically, A2aR inactivation was associated with decreased frequencies and reduced function of Foxp3^+^ Tregs, as evidenced by Foxp3 and CTLA-4 expression and classical effector T-cell proliferative assays, suggesting Treg modulation is a potential protective mechanism against sepsis. Simultaneously, the function and quantity of abdominal neutrophils were improved with ZM241385 treatment. To see if a link exists between them, Tregs and neutrophils were co-cultured, and it was found that ZM241385 blocked the inhibitory effect of Tregs on neutrophils. According to our research, Tregs play a key role in how A2aR antagonists improve sepsis prognosis and bacterial clearance.

## Introduction

Sepsis is a complicated condition that emerges as an abnormal host response to an infection and is linked to immediate organ failure and a high mortality risk (1). Due to the lack of specific drug therapy, sepsis mortality remains high (2, 3). More research on sepsis is required in the future.

Recently, researchers found that immunosuppression is a general immune condition in sepsis (4–6). In patients who die from sepsis, immunosuppression has been characterized as an increased number of regulatory T-cells (Tregs) (7). The majority of researches have concluded that the depletion of Tregs enhances bacterial clearance but does not benefit sepsis survival (8, 9). Many studies have indicated that the elimination of Tregs can induce a fatal autoimmune illness (10, 11), and this would be the reason why the depletion of Tregs enhances bacterial clearance but causes significant tissue damage in sepsis. Other studies suggest that modulating the suppressive functions of Tregs by silencing Foxp3 would benefit the sepsis model (12). However, there is still no effective method to moderately regulate Treg function in sepsis.

Recent research indicates that adenosine induces potent immunosuppression in sepsis *via* the adenosine 2a receptor (A2aR) (13, 14). Genetic depletion of the A2aR improved sepsis survival *via* bacterial clearance in several studies with an accompanying increase in bacterial clearance (15, 16). An A2aR antagonist (ZM241385) has been tested before and showed a similar protective effect (15); however, the mechanism of said A2aR antagonist has not yet been described. Previous research indicated that A2aR agonists could enhance Treg Foxp3, CTLA-4, CD39, and CD73 expression levels (17, 18). As described above, the immunosuppression activity of Tregs plays a critical role in sepsis. Thus, we hypothesize that the use of an A2aR antagonist is a proper method to inhibit the immunosuppression activity of Tregs, which could be a possible mechanism to improve sepsis survival.

A previous study described the influence of an A2aR antagonist on macrophages (15). As is known, enhancing macrophage function by granulocyte-macrophage colony-stimulating factor did not improve the survival rate of sepsis patients (19, 20). Here, we pay more attention to the effect of an A2aR antagonist on Treg function in a model of polymicrobial sepsis caused by cecal ligation and puncture (CLP). First, the A2aR antagonist ZM241385 was administrated to normal mice and Treg-depletion mice treated by PC61, an antibody of CD25. Then, co-culturing of Tregs and neutrophils was performed to explain the mechanism by which Tregs influence bacterial clearance. Our data indicated that ZM241385 improved sepsis survival by inhibiting Treg function, which inhibits bacterial clearance function of neutrophils.

## Materials and methods

### Animals

Male wild-type C57BL/6J mice aged 8–10 weeks (Beijing Vital River Laboratory Animal Technology Co., Beijing, China) were fed in a specific pathogen-free environment in Tianjin Medical University General Hospital Animal Laboratory (Tianjin, China). The average weight of the mice was 22-28g. All procedures involving the use of animals in research were authorized by the ethics committee of Tianjin Medical University General Hospital (approval no. IRB2022-DWFL-44). The mice were kept in an environment with a day/night cycle of 12 hours and had access to food and water at all times.

### Mouse model

The CLP mouse model was prepared as previously described (21). Mice were rendered unconscious using a gas anesthetic equipment and isoflurane. For the purpose of inducing sepsis, a 1-cm midline laparotomy incision was performed in the abdomen after disinfection; the cecum was then exteriorized, ligated in the center with 4-0 silk suture, and punctured with a 22-gauge needle at a spot 0.5cm from the cecal tip. The cecum was restored to the peritoneal cavity after a little drop of cecal content was expelled. Two layers of silk sutures were then used to seal the abdominal wound. After being revived with a subcutaneous injection of 1 mL of sterile saline (0.9% solution) into the scruff of the mice’s necks, the animals were kept warm on a thermal blanket and released back into their cages with unrestricted access to food and drink. The survival of one group of mice was examined every day for fourteen days. A second set of mice was re-anesthetized with isoflurane 24 hours following surgery, and their blood, peritoneal lavage fluid (PLF), and other organs were taken as shown below.

A lipopolysaccharide (LPS)-induced sepsis model was established *via* a single intravenous injection of LPS (1 mg/kg, O26:B6; Sigma-Aldrich, St. Louis, MO, USA) (22). In order to deplete Tregs *in vivo*, anti-CD25 antibody (100 μg, Clone PC61; BioLegend, San Diego, CA, USA) was injected intravenously 1 day before the CLP model was created (23). Separately, rat immunoglobulin (Ig)G (BioLegend) was injected into the control mice. The effect of Treg depletion was confirmed by flow cytometry. To test the A2aR antagonist effect, the mice were injected both immediately after the operation and 24 h thereafter with ZM241385 (5 mg/kg delivered subcutaneously) (MedChem Express, Shanghai, China) or normal saline.

### Collection of blood and PLF

After opening the chest, blood samples were taken aseptically by cardiac puncture, then placed on ice until further processing. The whole blood needed to be mixed with ethylenediaminetetraacetic acid EDTA (final volume, 4 mM) immediately to prevent coagulation. After completing successive dilutions for bacteriological examination (see below), the blood was centrifuged at 2000 ×g for 15 minutes, and the plasma obtained was kept at -80°C until further use.

The abdominal skin was cleansed with 75% ethanol before being opened to reveal the abdominal wall for peritoneal lavage. The peritoneal cavity was then filled with four milliliters of sterile physiological saline using a 22-gauge needle. After gently massaging the belly for one minute with the needle tip in the peritoneum, peritoneal fluid was extracted using the needle. When the retrieved PLF was ready to be processed for a bacteriological analysis, it was put on ice. The PLF was centrifuged at 1000 ×g for 10 min. after being serially diluted to determine colony-forming unit (CFU) counts (see below), and the supernatant was kept at -80°C until further analysis.

### Quantification of bacterial CFUs from PLF and blood

All samples of PLF and blood were prepared at 4°C environment and diluted with sterile phosphate-buffered saline (PBS), and 100 μL of each sample was spread on 5% sheep blood Trypticase™ soy agar (BD Bioscience, San Diego, CA, USA) plates, which were subsequently cultivated for 24 hours at 37°C in a damp chamber. The number of colonies was calculated and represented as CFUs per milliliter.

### Enzyme-linked immunosorbent assay (ELISA)

We utilized commercial ELISA kits in accordance with the manufacturer’s recommendations (Dakewe Biotech Co., Ltd., Shenzhen, China) to measure the concentration of interleukin (IL)-10, IL-6, and tumor necrosis factor (TNF)-a in the peripheral blood and the peritoneal cavity or the concentration of IL-10 and transforming growth factor (TGF)-β in the supernatant of Treg culture liquid, respectively.

### Isolation of cells from the peritoneal cavity

As previously described, peritoneal cells were collected from PLF (21). To keep peritoneal cells active, ice-cold PBS is installed into the peritoneal cavity instead of sterile physiological saline. Then, PLF was centrifugation at 300 ×g for 10 min at 4°C and washed twice with PBS. To prepare for flow cytometry, the pellet was suspended in 1 ml of cold PBS.

### Flow cytometry

Anti-mouse CD16/32 antibody was used to block Fc receptors on PLF or spleen cells by treating them with cold for 20 minutes. Single-cell suspensions were stained with a combination of the following antibodies: PerCP-anti-CD11b (M1/70), FITC-anti-Ly6G (1A8), PerCP-anti-CD4 (GK1.5), PE-anti-CD25 (PC61), FITC-anti-Foxp3 (FJK-16s), and APC-anti-CTLA-4 (UC10-4B9) (all from BioLegend). Neutrophils were recognized as CD11b^+^Ly6G^+^, and Tregs as CD4^+^CD25^+^Foxp3^+^. The cells were fixed and permeabilized following the kit’s instructions (BioLegend) before being stained for Foxp3. PerCP-conjugated rat IgG2b, PE-conjugated rat IgG2a, FITC-conjugated rat IgG2b, and APC-conjugated rat IgG1 were all used as isotype controls (all from BioLegend). Fluorescence-activated cell sorting (FACS) analysis was performed using an Accuir C6 Plus cell analyzer (BD Bioscience). Flow cytometry data were analyzed with FlowJo (Tree Star, Inc., Ashland, OR, USA). For all samples, ≥1×10^5^ cells were collected to generate scatter plots.

### Treg cell isolation

The splenocytes were isolated by passing spleen tissue through a cell strainer with a mesh size of 70 nm using the back of a sterile 3 mL syringe plunger. With the aid of red blood cell lysis buffer, RBCs were successfully removed. Single splenocytes were centrifugated at 300 ×g for 10 min at 4°C and washed twice with PBS. One milliliter of cold PBS containing 2% fetal bovine serum was used to suspend the pellet. Then, the cell suspension was incubated with a CD16/CD32 antibody to block the Fc receptors, and stained with CD4-PerCP and CD25-PE antibodies. For cell co-culture experiments, CD4^+^CD25^+^ Tregs were isolated by FACS using a FACSAria IIu instrument (BD Bioscience). The purity of Treg populations assessed by 2-color flow cytometry was >95%. As previously described (24), Tregs (5×10^4^-per well) were plated in 48-well plates precoated with CD3/CD28 antibodies (BD Bioscience) in RPMI 1640 supplemented with 10% fetal bovine serum, 1% penicillin-streptomycin (Sigma-Aldrich), and 2 mM of l-glutamine (Invitrogen, Carlsbad, CA, USA). Treg supernatant was collected after 24 h and cultured for TGF-β and IL-10 examination.

### Regulatory activity of Tregs

Similar to a previous study, we used the proliferation of carboxyfluorescein succinimidyl ester (CFSE) (Molecular Probes, Eugene, OR, USA)-labeled CD4^+^CD25^-^ T-cells (Tconvs) to measure the regulatory activity of Tregs (18). Briefly, cells were rinsed with PBS and treated with 1 μM of CFSE for 8 min to be labeled with CFSE at 37°C. Then, to remove any remaining CFSE, the cells were washed twice with fetal bovine serum. Tregs were isolated as described above from sepsis mice. Inhibition of effector T-cell proliferation was used to assess regulatory activity of Tregs. Tconvs were isolated from the spleens of C57BL/6 mice with the FACSAria IIu instrument and labeled with CFSE. Tconvs (1×10^5^ cells) were co-cultured with Tregs in a 1:1 ratio. Tconv cell proliferation was induced with anti-CD3 monoclonal antibody (1 μg/mL; BD Biosciences), anti-CD28 (1 μg/mL; BD Biosciences), and IL-2 (5 μg/mL; BD Biosciences) for 4 days in a 48-well plate, and after CD4+CD25- gating, the degree of Tconv proliferation was examined (25). In another set of experiments, ZM241385 (1 μM; MedChem Express) was added.

### Neutrophil isolation

Male mice aged 8 to 12 weeks had their femurs and tibias dissected. Before flushing the bone marrow cells from the tibias and femurs with ice-cold PBS containing 10% fetal bovine serum, we first cut the ends of each tibia and femur using dissecting scissors to expose the marrow. For tibias, we used two 1-mL quantities of buffer, and for femurs, we used three 1-mL volumes of buffer. By gently pipetting the combined bone marrow eluates back into suspension, we next carried out filtering using a 70-m nylon cell strainer (Becton, Dickinson, and Co., Franklin Lakes, NJ, USA) to remove cell clumps and bone particles. Using a centrifuge set at 300 g for 6 minutes at 4°C, the cell suspension was twice washed with PBS, and the pellet was then suspended in 1 mL of ice-cold PBS containing 4% fetal bovine serum. Then, the cell suspension was incubated with a CD16/CD32 antibody and stained with CD11b-PerCP and Ly6G-FITC antibodies. For cell co-culture experiments, CD11b^+^Ly6G^+^ neutrophils were isolated by FACS using the FACSAria IIu instrument. The purity of neutrophil cell populations assessed by 2-color flow cytometry was >95%.

### Co-culture of Tregs and neutrophil

As previously described (24), Tregs (5×10^4^ per well) were plated in 48-well plates pre-coated with CD3/CD28 antibodies (BD Bioscience) in RPMI 1640 supplemented with 10% fetal bovine serum, 2 mM of l-glutamine (Invitrogen), and 1% penicillin-streptomycin (Sigma-Aldrich). Then, neutrophils (5×10^5^ per well) were added to the co-culture at 37°C. After 5h, neutrophil phagocytosis and oxidative bursts were measured by flow cytometry analysis (see below). In another set of experiments, ZM241385 (1μM; MedChem Express) was added as described in previous research (26).

### Phagocytosis

Using a modified procedure, phagocytosis was evaluated by flow cytometry as previously mentioned (27). Briefly, 24 hours after CLP, peritoneal cells were collected, the red blood cells were lysed, and the cells were stained for neutrophil-specific surface markers. The stained cells were then incubated with opsonized PE-labeled *Escherichia coli* (K-12 strain) bioparticles (catalog no. E2861; Thermo Fisher Scientific, Waltham, MA, USA) for 10 min at 37°C, and analyses using flow cytometry were then carried out soon after.

### Oxidative bursts

As previously described, a modified procedure was used to measure the generation of reactive oxygen species (ROS) using flow cytometry (21). Briefly, at 24 hours post-CLP, peritoneal cells were collected. Following RBC lysis, peritoneal cells were loaded in the dark for 30 minutes at 37°C with 5 μM/mL of ROS Brite™ (10 µM; AAT Bioquest, Sunnyvale, CA, USA), which is a general oxidative stress indicator. As soon as the cells were washed and labeled for neutrophil-specific surface markers, flow cytometry analysis was done.

### Statistical analysis

All statistical comparisons were analyzed by the GraphPad Prism software program (GraphPad Software, San Diego, CA, USA). The log-rank test was used to examine the survival curves. In order to compare cytokine levels, CFUs, and other lab variables, we employed two-tailed *t* testing. For all experiments, *p* < 0.05 was accepted as statistically significant. Data are shown as mean ± standard error of the mean values unless otherwise noted.

## Results

### The A2aR antagonist improved sepsis survival *via* bacterial clearance

**Figure 1:**
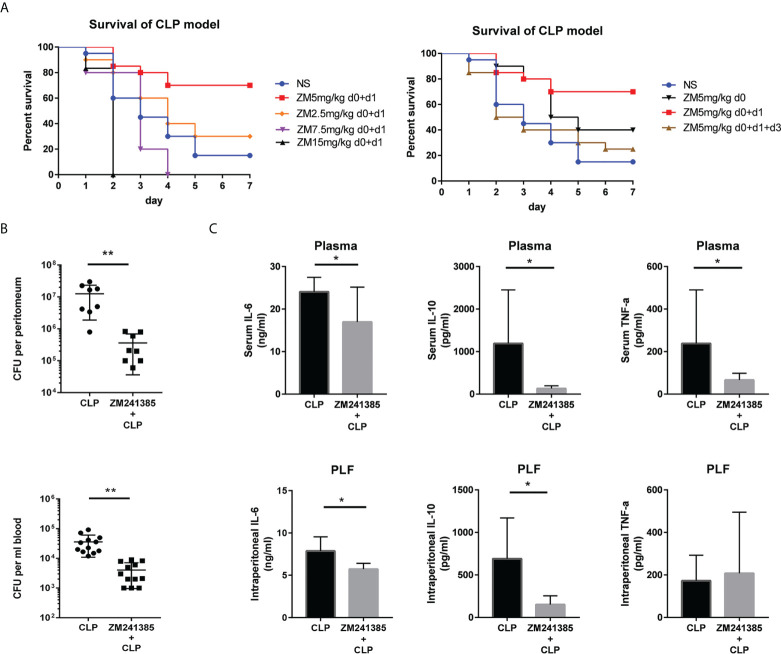
ZM241385 protects against CLP-induced mortality with reduced cytokine storm and improved bacterial clearance. Mice were subjected to CLP or ZM241385+CLP (n = 30 per group). Following CLP, the survival rates were assessed for a continuous 7 days. **(A)** Administration of 5 mg/kg of ZM241385 for 2 days led to a better effect than other doses in C57BL/6J mice. Statistical differences were determined by the log-rank test. **(B)** PLF and peripheral blood were taken 24 hours after CLP (n = 8-11 per group). Bacterial CFUs were calculated using 100 μL of each sample after dilution on Trypticase™ soy agar blood plates. ZM241385-treated mice exhibited a lower bacterial burden. **(C)** Mice treated with the selective A2aR antagonist ZM241385 had lower levels of IL-10 and IL-6 in their plasma and peritoneum (5 mg/kg, delivered subcutaneously once daily). Comparing animals treated with ZM241385 to mice treated with a vehicle, TNF-α levels in the blood were reduced. Levels of IL-6, IL-10, and TNF-α were measured by ELISA in plasma and PLF obtained 24h after CLP. Data are presented as mean ± standard error of the mean values of n = 7-10 mice per group. These findings are typical of three independent trials. The individual mice are represented by symbols. *p < 0.05, **p < 0.01.

A clinically relevant model of polymicrobial peritonitis caused by CLP that has been widely used to study sepsis was employed to establish severe sepsis in mice (28, 29). We tested different kinds of administration of ZM241385 and found the proper method to improve sepsis survival (**Figure 1A**). Bacterial clearance has been wildly used to evaluate sepsis and corelates with survival (13, 15). We found that the ZM241385-treated sepsis group experienced higher bacterial clearance from both the blood and peritoneal cavity (**Figure 1B**). Cytokine storm is another important indicator for sepsis survival (4). We found that IL-6, IL-10 and TNF-α levels of the ZM241385-treated sepsis group were decreased in the blood and peritoneal cavity (**Figure 1C**).

**Figure 2:**
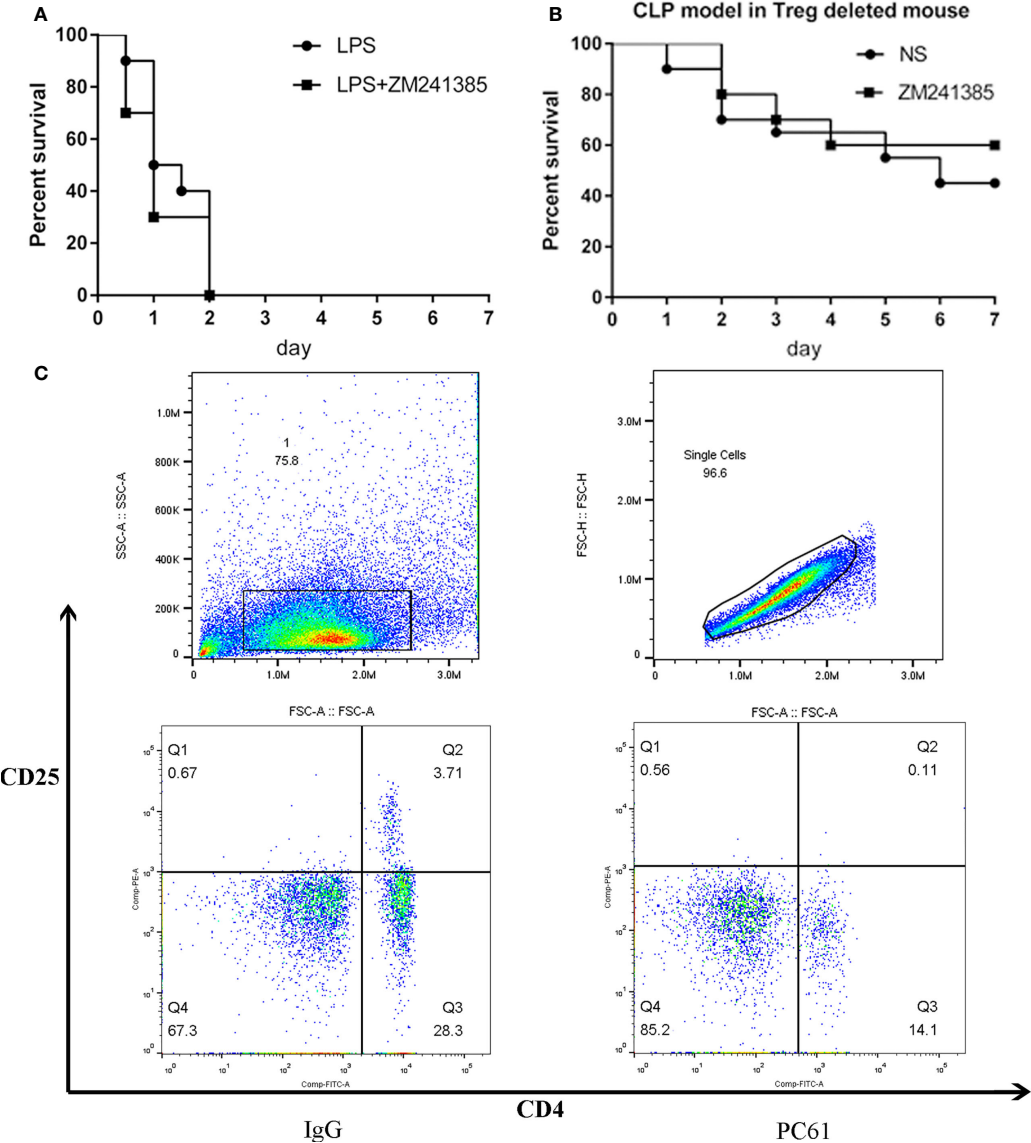
ZM241385 (5 mg/kg) did not improve LPS-induced sepsis survival and CLP-induced sepsis survival in Tregs deleted mice. **(A)** The LPS induced sepsis model was administrated by a single intravenous injection of LPS (1 mg/kg). Mice were subjected to LPS or ZM241385+LPS (n = 10 per group). The survival rates were evaluated for a consecutive 7 days after CLP. **(B)** To deplete Tregs in vivo, 100 μg of anti-CD25 antibody (Clone PC61) was injected intravenously one day before the CLP model. Rat IgG was injected into the control mice. The effect of deletion is confirmed by Flow Cytometry. Septic mice were subjected to normal saline (NS) or ZM241385 (n = 10 per group). **(C)** The survival rates were evaluated for a consecutive 7 days after CLP. A statistical difference was determined by the log-rank test.

In order to test whether an A2aR antagonist could improve sepsis survival by decreasing system inflammation, LPS was used to establish endotoxemia in mice (30). Even using several different administrations of ZM241385, survival from LPS-induced endotoxemia did not improve (**Figure 2A**). In several studies, Tregs show abilities to inhibit bacterial clearance (31–33). PC61 was used to achieve short-term depletion of Tregs (23, 34), and the result of depletion was confirmed by flow cytometry (**Figure 2C**). Then, CLP model creation and administration of ZM241385 proceeded in the Treg-deleted mice. Ultimately, we found that ZM241385 did not benefit sepsis survival (**Figure 2B**).

### The A2aR antagonist inhibited Treg activity and enhance neutrophil functions in sepsis

**Figure 3:**
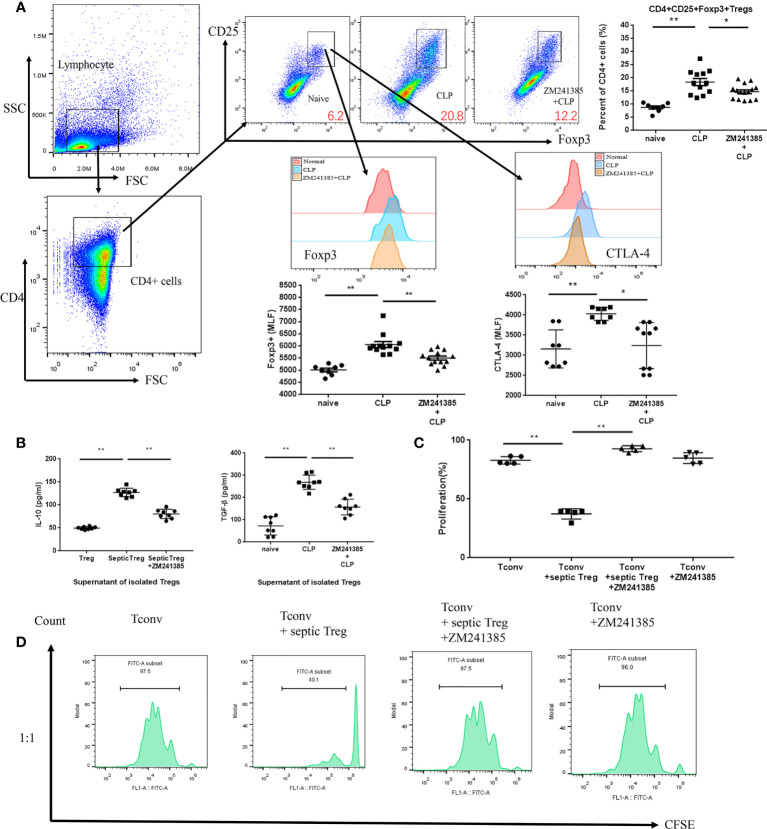
A2aR blockade treatment modulates the frequency and function of Tregs in CLP mice. Tregs were collected for testing from the spleens of normal, CLP and ZM241385-treated CLP mice. Tregs were identified by CD4+CD25+Foxp3+ to elevate percentages of CD4+ cells. **(A)** Percentages of Tregs from ZM241385-treated CLP mice were decreased compared to those from the CLP group. Expression levels of Foxp3 and CTLA-4 in Tregs were measured by flow cytometry. Representative histograms and the mean fluorescence intensity (MFI) of Foxp3 and CTLA-4 expression are represented. **(B)** Tregs from the CLP group secreted more cytokines than those from the ZM241385-treated group and naïve group. **(C, D)** Treg suppression of T-cell proliferation might be reduced if the A2aR was blocked. The individual mice are represented by symbols. *p  < 0.05, **p  < 0.01.

**Figure 4:**
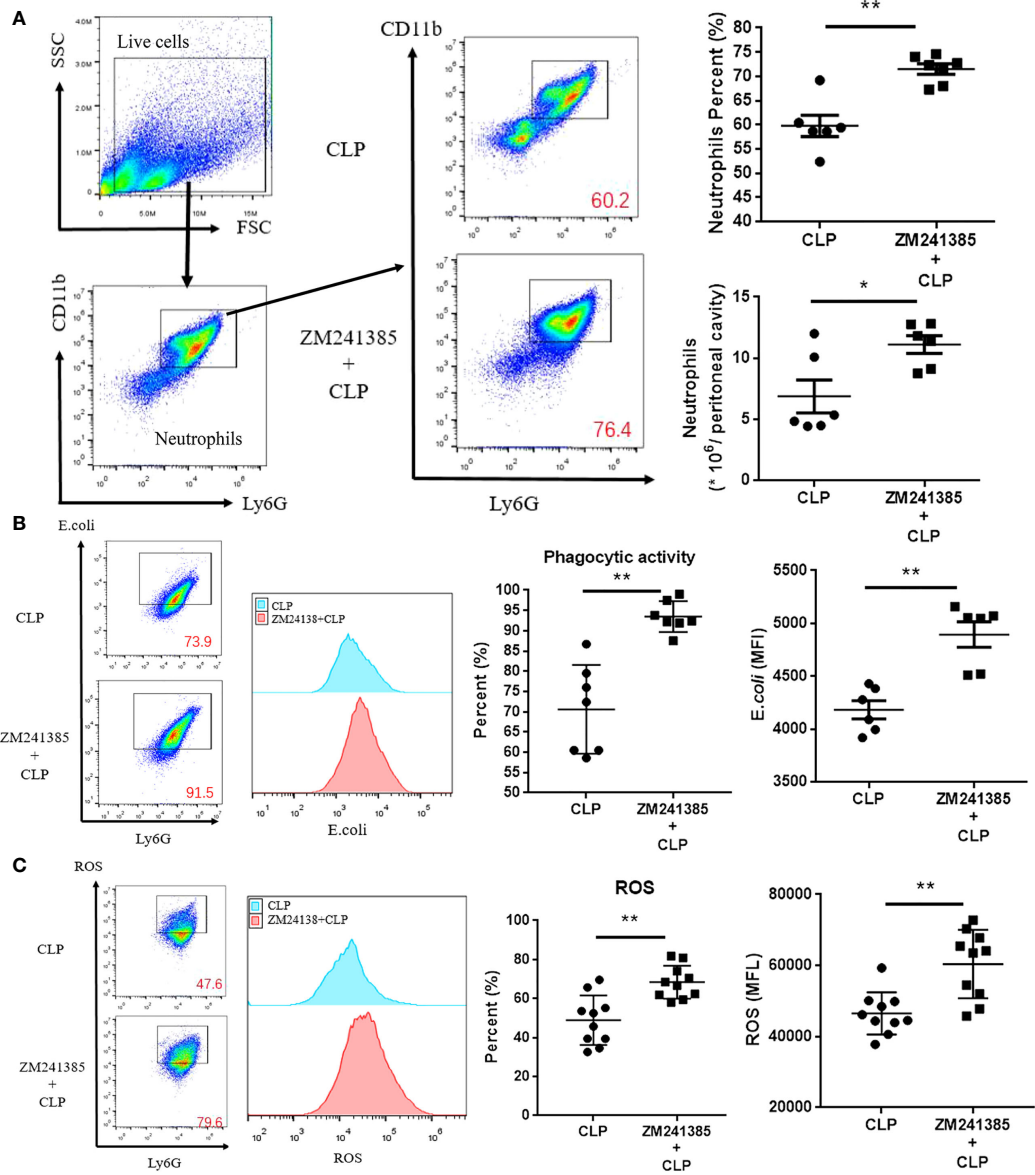
ZM241385 improves neutrophil phagocytosis and ROS generation in CLP-induced sepsis. Mice treated with vehicle or ZM241385 were collected for peritoneal cells 24 hours after CLP. Neutrophils were identified by Ly6G+CD11b+ in elevated numbers and percentages **(A)** and were assessed for phagocytic capacity **(B)** or ROS production **(C)**. In Fig. 3B and Fig. 3C panels, CD11b+ and Ly6G+ cells were gated first, as in Fig. 3A. After that, all the cells in Fig. 3B and Fig. 3C were Ly6G+ and considered neutrophils. The Y axis of these panels is used to describe the function of neutrophils. The function of neutrophils is normal distribution. We make the same mistake in each test by drawing the rectangular box as the positive groups. Meanwhile, the fluorescence curve was used to show the difference between each group. The individual mice are represented by symbols. *p  < 0.05, **p  < 0.01.

In sepsis, Treg activity is enhanced, which is characterized by increased expression levels of CTLA-4 (35, 36) and Foxp3, higher IL-10 and TGF-β production, and greater suppression of T-cell proliferation (37). We found that the A2aR antagonist ZM241385 inhibited Treg expansion, Foxp3 and CTLA-4 (**Figure 3A**) expression, and IL-10 or TGF-β secretion (**Figure 3B**). The inhibition of T-cell proliferation has been tested to describe Treg inhibition activity in several studies (38, 39). We found that the A2aR antagonist ZM241385 inhibited Treg inhibitory activity on T-cell proliferation (**Figures 3C, D**). Reduced neutrophil antimicrobial activity has been linked to increased mortality in sepsis (40, 41). We found that neutrophils in PLF increased in ZM241385-treated sepsis mice (**Figure 4A**), and neutrophil phagocytosis (**Figure 4B**) and ROS production (**Figure 4C**) were similarly enhanced in this group.

### Inhibition of Treg activity by the A2aR antagonist is the reason for neutrophil function enhancement in sepsis

**Figure 5:**
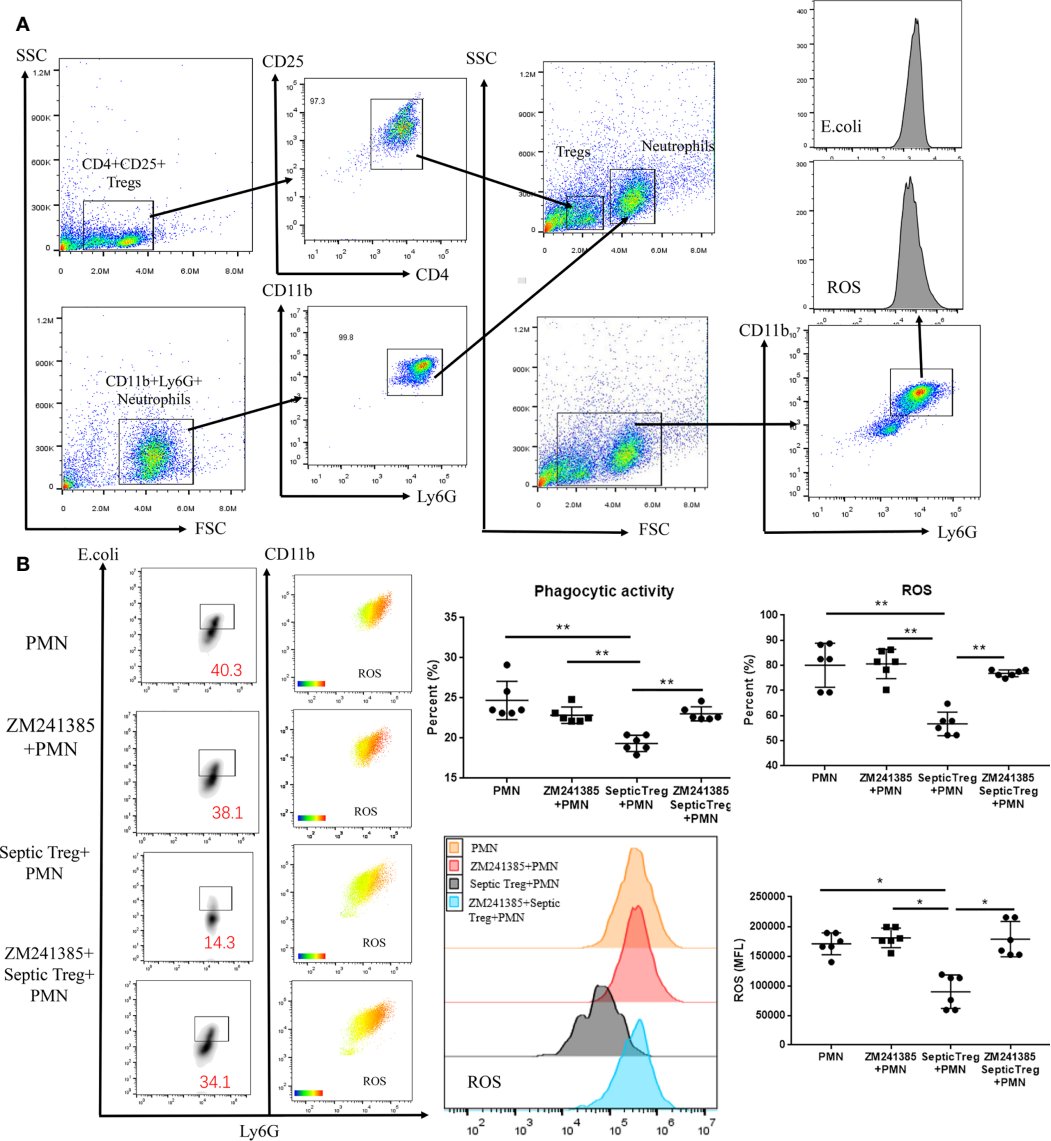
ZM241385 influenced Treg-mediated inhibition of neutrophil phagocytosis and ROS generation in vitro. CD4+CD25+ Tregs and CD11b+Ly6G+ neutrophils were isolated by FACS using the FACSAria IIu instrument. The purity of Treg and neutrophil populations assessed by 2-color flow cytometry was >95%. **(A)** Neutrophils were co-cultured with septic CD4+CD25+ Tregs and their phagocytic capacity or ROS generation ability was evaluated as previously described. **(B)** We found that neutrophil phagocytosis and ROS generation could be inhibited by septic Tregs and restored by ZM241385. *p  < 0.05, **p  < 0.01.

Previous research has indicated that Tregs inhibit neutrophil functions in several mechanisms (24, 42). Co-culture of Tregs and neutrophils has been wildly used to reveal the inhibitory effect of Tregs on neutrophils (43, 44). In order to prove whether the A2aR antagonist ZM241385 enhanced neutrophil function *via* a Treg-dependent mechanism in sepsis, we performed a co-culture of Tregs and neutrophils **(** **Figure 5A** **)** in different conditions and found the following results **(** **Figure 5B** **)**. First, ZM241385 did not enhance neutrophil phagocytosis or ROS generation function. Then, we found that Tregs from sepsis mice greatly inhibited neutrophil phagocytosis and ROS generation function. Finally, ZM241385 enhanced neutrophil phagocytosis and ROS generation function in the co-culture of Tregs and neutrophils. In summary, the inhibition of Treg activity by an A2aR antagonist is the mechanism for enhanced neutrophil function in sepsis.

## Discussion

Adenosine is one of the oldest organic molecules on Earth, having appeared > 100 million years before the birth of the first living form (45, 46). Pharmacology intervention of A2aRs has been used clinically in several conditions; for example, an A2aR agonist has been used in myocardial perfusion imaging (47), and an A2aR antagonist has been used in Parkinson’s disease (48, 49). In this study, we used an A2aR antagonist for the treatment of polymicrobial sepsis and found that the survival of our CLP mouse model was improved by ZM241385. In contrast, ZM241385 showed no benefit in LPS-induced endotoxemia. It is the associated enhanced bacterial clearance but light tissue damage that is the major beneficial effect of an A2aR antagonist to improve sepsis survival. Consistently, an A2aR agonist paired with antibiotics protected against sepsis created by *E. coli* injection by inhibiting the excessive inflammatory reaction caused by the rapid drug-mediated death of vast numbers of bacteria (50).

Immunosuppression in sepsis has been linked to Tregs and myeloid-derived suppressor cells (51). Here, we paid more attention to Tregs because prior research has shown that both adenosine and Treg levels rise in the blood of sepsis patients (51, 52). Some studies have indicated that A2aR agonists can enhance the expression levels of Foxp3, CTLA-4, CD39, and CD73 in Tregs (17, 18). Whether or not an A2aR antagonist inhibits Treg function has not been studied. We found in sepsis that the A2aR antagonist ZM241385 inhibited Treg expansion, CTLA-4 and Foxp3 expression, secretion of IL-10 and TGF-β and inhibitory activity on T-cell proliferation. In detail, ZM241385 seems to mostly target CTLA-4 expression, whereas its effect on Treg numbers or FoxP3 expression is moderate. This is consistent with what was found in earlier studies, which showed that the sepsis model would be helped by adjusting the suppressive functions of Tregs (12) but not by getting rid of them (8, 9). In addition, we cautiously found that ZM241385 did not improve sepsis survival without Tregs. In summary, an A2aR antagonist improved sepsis survival by blocking the Treg-induced inhibition of bacterial clearance.

Both neutrophils and macrophiles can clear bacteria in sepsis (53, 54). A previous study indicated that an A2aR antagonist enhanced macrophage functions (15). In this study, we found that neutrophil count and the activities of phagocytosis and ROS production in PLF were enhanced by the A2aR antagonist ZM241385. As neutrophils hold an essential role of pathogen control (55, 56) and neutropenia is a major reason for fatal infection (50), we did not examine the role of neutrophils in bacterial clearance by depletion of neutrophils.

Previous research has demonstrated that Tregs may inhibit neutrophil ROS generation and promote neutrophil apoptosis and death through many mechanisms, including the contact-dependent CTLA-4/B7-1 route and the IL-10 impact (24, 42). Here, we validated the idea that Tregs from sepsis mice strongly inhibited neutrophil phagocytosis and ROS production. Differently, we found that the A2aR antagonist ZM241385 could reduce the inhibitory activity of Tregs on neutrophils. As it could not affect neutrophil function alone, our data indicate that the A2aR antagonist ZM241385 just interfered with the inhibitory activity of Tregs in sepsis.

There are also some imperfections in our study. First, the fact that PC61 was used to delete Tregs may influence some CD25^+^ effector T-cells (57). However, PC61 has been used in several studies to delete Tregs in the short term (23, 34). Importantly, we preferred to find the effect of an A2aR antagonist in a model with a normal gene background, which is more similar to the typical clinical condition. Second, we found that the A2aR antagonist ZM241385 could inhibit Treg function in sepsis, but we did not test the molecule mechanisms of A2aR signal enhancement of Treg activity. These mechanisms must be investigated in future studies.

Collectively, our data suggest that an A2aR antagonist would be a proper treatment to inhibit Treg activity in sepsis and consequently enhance neutrophil phagocytosis and ROS generation function to enhance bacterial clearance and improve the survival rate. More research is needed to describe the intracellular pathway of Tregs to gain a better understanding of immunosuppression in sepsis.

## Data availability statement

The original contributions presented in the study are included in the article/supplementary material. Further inquiries can be directed to the corresponding author.

## Ethics statement

The animal study was reviewed and approved by the ethics committee of Tianjin Medical University General Hospital.

## Author contributions

TM and TZ contributed to the conception and design of the study. TM organized the database. TZ performed the statistical analysis. TZ and JZ wrote the first draft of the manuscript. JZ, JF, GC, and TZ wrote sections of the manuscript. TZ, JZ, JF, and GC performed experiments. All authors contributed to manuscript revision and have read and approved the submitted version.

## Funding

This work was supported by grants from the National Natural Science Foundation of China (82172122 to TM), and Tianjin Municipal Health Commission (RC20145 to TZ). This work was also funded by the Tianjin Key Medical Discipline (Specialty) Construction Project.

## Acknowledgments

We thank Liu Ye (Department of Neurology, Tianjin Medical University General Hospital, Tianjin, China) and Li Hong-Yue (Department of General Surgery, Tianjin Medical University General Hospital, Tianjin, China) for their technical assistance.

## Conflict of interest

The authors declare that the research was conducted in the absence of any commercial or financial relationships that could be construed as a potential conflict of interest.

## Publisher’s note

All claims expressed in this article are solely those of the authors and do not necessarily represent those of their affiliated organizations, or those of the publisher, the editors and the reviewers. Any product that may be evaluated in this article, or claim that may be made by its manufacturer, is not guaranteed or endorsed by the publisher.
